# Effect of Composting on Dissolved Organic Matter in Animal Manure and Its Binding with Cu

**DOI:** 10.1100/2012/289896

**Published:** 2012-10-17

**Authors:** Fengsong Zhang, Yanxia Li, Xiong Xiong, Ming Yang, Wei Li

**Affiliations:** ^1^State Key Laboratory of Water Environment Simulation, School of Environment, Beijing Normal University, Beijing 100875, China; ^2^State Key Laboratory of Environmental Criteria and Risk Assessment, Chinese Research Academy of Environmental Sciences, Beijing 100012, China

## Abstract

The agricultural application of raw animal manure introduces large amounts of dissolved organic matter (DOM) into soil and would increase transport of heavy metals such as Cu which are widely present in animal manure. The purpose of this research was to evaluate the evolution of DOM from pig and cattle manures during composting through excitation-emission matrix (EEM) fluorescence spectroscopy and the binding ability of DOM toward copper (Cu) ions with the aid of fluorescence quenching titration. The excitation-emission matrix spectra indicated that tyrosine-like, tryptophan-like, and soluble microbial byproduct-like fluorescence decreased significantly, while humic-like and fulvic-like fluorescence increased and became the main peaks in composted manure DOM. Fluorescence quenching titration showed that the complexing capacities of pig and cattle manure DOM decreased after composting. Correlation analysis confirmed that complexing capacity of DOM positively and significantly correlates with tyrosine-like and soluble microbial byproduct-like materials which mostly degraded after composting. These results would suggest that the ability of manure DOM to complex with Cu is inhibited as a result of reduced protein-like materials after composting.

## 1. Introduction

Animal manure was usually applied to arable soils in order to improve soil fertility and increase the organic matter content. However, in recent years, high concentration of heavy metal such as Cu in animal manure has been frequently reported in China due to abuse of mineral additives [1, 2]. Because part of the organic substances in animal manure are water soluble, a direct impact of the application of animal manure to agricultural land is the release of dissolved organic matter (DOM) into soil solution [3]. DOM could complex with heavy metals and then improve their transport to surface water [4, 5].

In natural water, humic acids and fulvic acids are major components and represent up to 70% of DOM, which contributed the most organic ligands to Cu complexing [6]. However, a recent study indicates that nonhumic substances such as amino acids are likely engaged in the Cu complexation [7]; as one type of protein-like materials, amino acids are important components in DOM of organic wastes [8–10]. In addition, de Zarruk et al. [9] verified that the fraction in vinasse with the highest proteinaceous fluorescence has the greatest ability to bind with Cu. According to previous research, raw pig manure and cattle manure DOM also have a large number of proteinaceous materials [8, 10]. Whether the proteinaceous materials in manure DOM play a key role in complexing with Cu was not clear.

Composting is a useful method for organic wastes stabilization [11–13]. A decline in DOM has been reported by Inbar et al. [14] and Huang et al. [12] for cattle manure and pig manure composting, respectively. The common characteristic is that DOM composition may undergo significant transformation after the composting process [10, 15]. For example, domestic organic wastes (coffee residues and garden trimmings) had a reduction of carbohydrates and increase of aromatic, phenolic, carboxylic, and carbonylic C in DOM after composting [16]. Accordingly, a decrease in the tyrosine-like and tryptophan-like materials and an increase in the humic, and fulvic-like materials were observed by excitation-emission matrix (EEM) fluorescence spectroscopy during composting of winery residues and municipal solid wastes [10, 13]. However, little is reported about the transformation of animal manure DOM during composting process at present. Furthermore, variation of binding ability of manure DOM with heavy metals after composing was also unknown. 

As a selective, sensitive, and nondestructive analytical technique, EEM fluorescence spectroscopy has been always used to characterize the DOM composition using contour plots, number of fluorescence peaks, position of wavelength-independent fluorescence maxima (Ex_max_/Em_max_), and fluorescence intensity at Ex_max_/Em_max_ [8, 17–19]. However, it is limited to quantifying the properties based on one, two, or three data points from the fluorescence spectra. Chen et al. [20] developed the fluorescence regional integration (FRI) technique for quantitative analysis, which has been successfully used to study the evolution of organic waste DOM during the composting process [10, 13]. In addition, fluorescence spectroscopy has been revealed as a very promising technique for the study of metal ion binding to DOM [7, 21, 22]. Metal ions, especially paramagnetic metals, that is, Cu and Hg, are able to quench the intrinsic fluorescence of DOM [23, 24]. Therefore, together with the fluorescence quenching titration, the metal ion complexing capacities of DOM and stability constants of metal-DOM complexes can be examined. 

The objectives of this study were (1) to explore the composition evolution of animal manure (pig manure and cattle manure) DOM during composting by EEM fluorescence spectroscopy; (2) to investigate the effect of composting process on manure DOM complexation with Cu.

## 2. Materials and Methods

### 2.1. Composting Procedure and Sample Preparation

The composting experiment was conducted in the same way with our previous study [22]. Manures and bulking agents were collected at local farms. Sawdust and corn stalks were chopped into 2-3 cm pieces and air dried before composting. The treatments of the composting piles on a dry volume basis were as follows. Treatment A: 50% pig manure + 50% sawdust; treatment B: 50% pig manure + 50% corn stalks; treatment C: 50% cattle manure + 50% sawdust; treatment D: 50% cattle manure + 50% corn stalks. 

The composting experiments were performed in cylindrical vessels (diameter: 500 mm; height: 600 mm). The uniform forced ventilation was equipped at a rate of 0.1 m^3^/min for 10 minutes at 60-minute intervals through perforated plates fixed at the bottom of the vessels to provide oxygen. The moisture content of each pile was kept at 50–60% (weight/weight) during composting. In the first 30 days of composting, the piles were turned periodically to keep the temperature under 60°C. Afterwards, the forced ventilation was stopped, and the piles were stirred daily for further humification. 

The composting process was stopped when the compost temperature equaled the ambient temperature with no measurable changes for approximately 20 days. The pig manure and cattle manure were composted for 71 days and 46 days, respectively. Samples were collected from treatment A and treatment B on days 1, 8, 11, 17, 32, and 71 (A1, B1, A8, B8, A11, B11, A17, B17, A32, B32, A71, and B71), whereas sampling was done on days 1, 6, 10, 13, 29, and 46 (C1, D1, C6, D6, C10, D10, C13, D13, C29, D29, C46, and D46) for treatment C and treatment D, respectively. The subsamples were taken at different positions within the vessel and then thoroughly mixed as a composite sample. Prior to extracting the DOM, the samples were air dried.

### 2.2. Extraction of DOM and Fluorescence Analysis

Two grams of subsamples were extracted with 40 mL of deionized water and shaken for 24 hours. The solution was then centrifuged at 10,000 rpm for 10 minutes. The supernatant was then filtered using Whatman GF/F glass microfiber filter papers that had previously been heated at 450°C to remove any possible organic matter. The extracts were immediately analyzed for dissolved organic carbon using the TOC analyzer (Liqui TOC, Elementar, Germany).

The fluorescence of the filtered DOM samples was determined with a model F-4500 fluorescence spectrophotometer (Hitachi, Japan) with a 150-W Xe arc lamp. Prior to fluorescence analysis, all sub-samples for fluorescence analysis were diluted to the uniform concentration of 10 mg C/L to reduce inner filter effects [9]. To generate an EEM, excitation wavelengths were scanned from 200 to 400 nm in 2 nm steps, and the emitted fluorescence was detected between 300 and 550 nm in 5 nm steps. The band-pass width was 5 nm for excitation and 10 nm for emission, and the scan speed was 2400 nm/min [24]. 

Fluorescence regional integration method was applied for spectral comparison to thoroughly explore the transformation of DOM composition during manure composting [20]. To avoid the scattering effects of fluorescence data, the treatment method of the first-order Rayleigh, Raman and second-order Rayleigh scatters was applied, which was proposed by Bahram et al. [25]. The EEM plots were generated from the fluorescence spectral data using Sigmaplot 10.0 software (Systat Software, Inc.).

### 2.3. Fluorescence Quenching Titration and Complexation Modeling

Fluorescence quenching titration was carried out to characterize the complexation of manure DOM with Cu according to the research of Plaza et al. [21]. Experiments were carried out by adding 0.01 M Cu(NO_3_)_2_ solutions to a series of glass bottles that contained 50 mL of DOM solution. The pH value was then adjusted to 7.0. All samples were shaken in the dark for 24 hours under a nitrogen atmosphere at constant temperature (25 ± 0.1°C) to ensure complexation equilibrium.

The selection of the wavelengths for the fluorescence titration was based on the highest fluorescence intensity observed from the EEM of the samples [21]. The complexation model of Ryan and Weber was used to determine the binding parameters between DOM and Cu ions [26]. The model assumes a simple 1 : 1 equilibrium between a metal ion and an organic ligand

(1) I=I0+(ICuL−I0)(12KCuCL)×(1+KCuCL+KCuCCu  −(1+KCuCL+KCuCCu)2−4KCu2CCuCL),

where *I* and *I*_0_ are the fluorescence intensity (arbitrary units) at the Cu concentration of *C*_Cu_ and at the start of the titration, respectively, *I*_Cu*L*_ is the limiting value below which the fluorescence cannot decrease with the addition of Cu^2+^, *C*_*L*_ is the total ligand concentration, *C*_Cu_ is the total Cu ion concentration, and *K*_Cu_ is the conditional stability constant.

The complexation capacity (CC_Cu_), that is, the amount of active binding sites per unit mass of DOM, was calculated as

(2) $$\begin{array}{c} \text{C}{\text{C}}_{\text{Cu}}=\frac{C_{L}}{{\left(\text{DOM}\right)}_{\text{total}}}, \end{array}$$

where (DOM)_total_ is the total concentration of DOM. *K*_Cu_ and *C*_*L*_ were solved by a nonlinear regression analysis with the software 1st Opt 1.5 (7D-soft High Technology Inc., China). The optimum set of fitting parameters for each DOM sample was obtained by iteratively varying the adjustable parameter values until the sum of the squares of the differences between the observed and fitted values of *I* was minimized. Full, unconstrained optimization was achieved using the quasi-Newton algorithm. 

### 2.4. Statistical Analysis

Correlations were analyzed between percentages of fluorescence response (*P*_*i*,*n*_) to elucidate the transformation of DOM during the composting. Meanwhile, correlation analysis was also used to determine the correlations between *P*_*i*,*n*_ and parameters of DOM binding with Cu (i.e., CC_Cu_ and log⁡⁡*K*_Cu_). Statistical analyses were performed with the software SPSS 11.5 (SPSS Inc., Chicago, IL, USA) for Windows. 

## 3. Results and Discussion 

### 3.1. Change of DOM Concentrations during Composting

**Figure 1:**
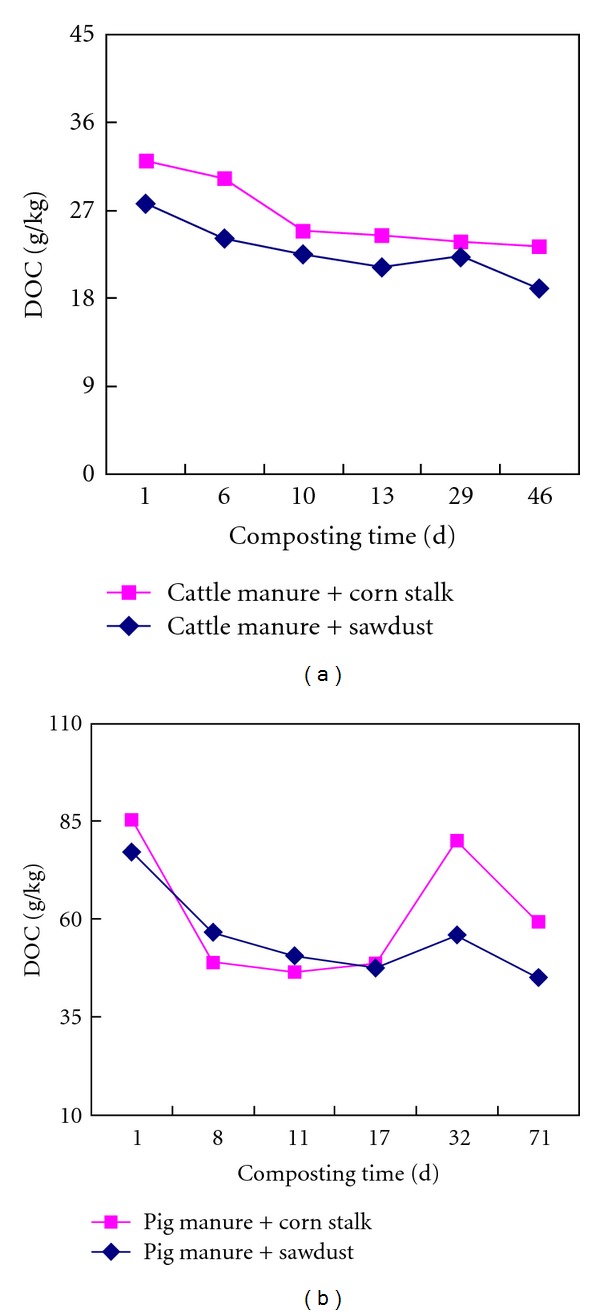
Changes of dissolved organic matter (DOM) concentrations during manure composting.

The total concentration of DOM in pig and cattle manure was reduced after composting (Figure 1), which was similar to results obtained by Inbar et al. [14] and Huang et al. [12]. Cattle manure DOM concentrations continuously declined during the composting process, with a sharp reduction occurring in the initial stage of composting. During co-composting with corn stalk and sawdust, DOM in cattle manure was reduced by 27.4% and 31.4%, respectively. However, during cocomposting with exhausted grape marc, cattle manure DOM only reduced by 18.3% [27].

During composting of pig manure, DOM increased after a sharp initial decrease. At the end of the composting process, DOM concentration in pig manure with addition of sawdust and corn stalk decreased to 58.6% and 69.5%, respectively, of the raw materials after composting. In comparison to the cattle manure, there is more DOM degraded during pig manure composting than cattle manure, using the same composting method. This observation may reflect that pig manure contains far more DOM than cattle manure, but that the DOM in pig manure is also more easily degradable. In contrast, it was reported that about 95.8% of DOM in municipal solid waste had been degraded at the end of composting [13]. Our results and the cited study show that the rate of decrease in DOM concentration depends not only on the composting technique utilized, but also on the composition of the source material.

### 3.2. DOM Fluorescence Characteristics

**Figure 2:**
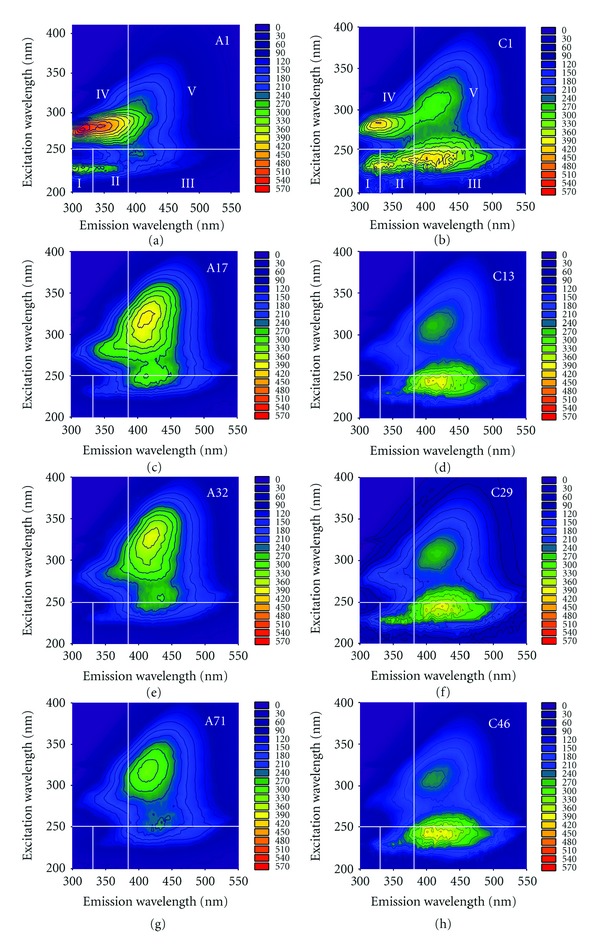
Excitation-emission matrix (EEM) spectra of DOM from treatment A (pig manure + sawdust) on days 1, 17, 32, and 71 (A1, A17, A32, and A71) and treatment C (cattle manure + sawdust) on day 1, 13, 29, and 46 (C1, C13, C29, and C46).

As the EEM spectra evolution of DOM from treatment B and treatment D were similar to that from treatment A and treatment C, the DOM spectra of treatment A and treatment C are displayed as the representative in Figure 2. According to the research of Chen et al. [20], the fluorescence of regions I, II, and IV in manure DOM are related to tyrosine-like, tryptophan-like, and soluble microbial byproduct-like materials while the fluorescence of regions III and V are related to fulvic-like and humic-like materials. Soluble microbial byproduct-like materials also contained another kind of tyrosine-like and tryptophan-like compounds, which were different from materials associated with region I and region II [28]. In the raw pig manure DOM (A1), the most intense fluorescence peak of Ex/Em = 280 nm/342 nm centered at region IV. Protein-like fluorescence peaks such as tyrosine-like and tryptophan-like peaks were associated with growth of living organisms in marine water [17]. However, these peaks were also detected in organic wastes, such as animal slurry and landfill leachates [8, 29]. In addition, we observed a peak of Ex/Em = 245 nm/399 nm centered at region III in raw pig manure DOM (A1), which may be attributed to aromatic and aliphatic groups in the DOM and widely labeled as fulvic-like substances [19]. However, there was no obvious humic-like fluorescence peak in pig manure DOM. Similar to raw pig manure, cattle manure DOM (C1) had intense tryptophan-like and tyrosine-like fluorescence peaks. However, cattle manure also had two intense fulvic acid-like and humic acid-like peaks centered at Ex/Em = 245 nm/400 nm and 305 nm/412 nm.

**Figure 3:**
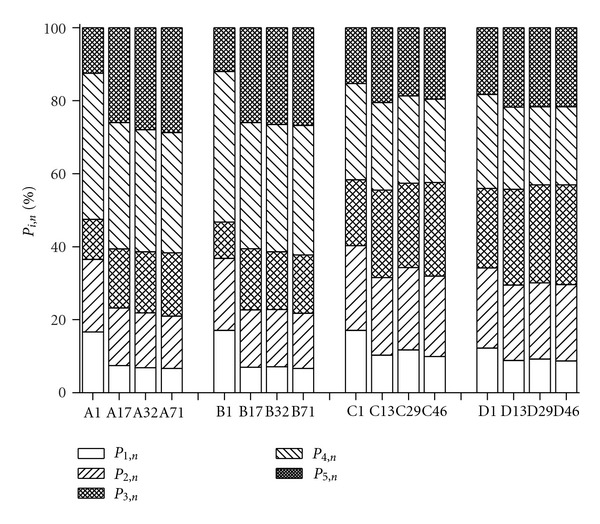
Evolution of the percentage fluorescence response (*P*_*i*,*n*_) during composting.

The percentage fluorescence response (*P*_*i*,*n*_) of the five regions in the different composting samples is displayed in Figure 3. In the raw pig manure DOM (A1), the quantities of *P*_1,*n*_, *P*_2,*n*_, and *P*_4,*n*_ in raw pig manure were approximately 16.6%, 19.9%, and 40.0% of total DOM, respectively, while the fulvic-like, and humic-like materials only contribute 10.9%, and 12.5%, respectively. In contrast, *P*_*i*,*n*_ of tyrosine-like, tryptophan-like and soluble microbial byproduct-like materials in cattle manure DOM were 17.1%, 23.2%, and 26.3%, while the fulvic acid-like and humic acid-like substances were 18.1% and 15.3%. Thus, there are more humic substances in cattle manure DOM than in pig manure DOM in the manures obtained for this study. 

DOM from treatment A (pig manure + sawdust) and treatment B (pig manure + corn stalk) was on days 1, 17, 32, and 71 (A1, B1, A17, B17, A32, B32, A71, and B71), treatment C (cattle manure + sawdust) and treatment D (cattle manure + corn stalk) on days 1, 13, 29, and 46 (C1, D1, C13, D13, C29, D29, C46, and D46). 

During the composting process, a sharp decrease of tyrosine-like, tryptophan-like, and soluble microbial byproduct-like fluorescence occurred in the initial stage of composting for pig manure and cattle manure, while fulvic-like and humic-like fluorescence displayed reverse trends. At the end of composting, there were only fulvic-like and humic-like fluorescence peaks left both in cattle manure and pig manure DOM. The total *P*_5,*n*_ values of pig manure or cattle manure cocomposting with sawdust increased by 16.3% and 4.2%, respectively, compared with the raw materials, while the values of *P*_3,*n*_ increased by 6.4% and 7.6%, respectively. Similar results were obtained for DOM evolution of exhausted grape marc cocomposted with cattle manure and poultry manure in the study of Marhuenda-Egea et al. [10]. Shao et al. [13] also reported that *P*_5,*n*_ increased from 18.8% to 54.8%, and humic acid-like compounds became the main component in municipal solid wastes DOM at the end of the composting process.

### 3.3. Implications of the DOM Change on Cu-DOM Complexation

**Figure 4:**
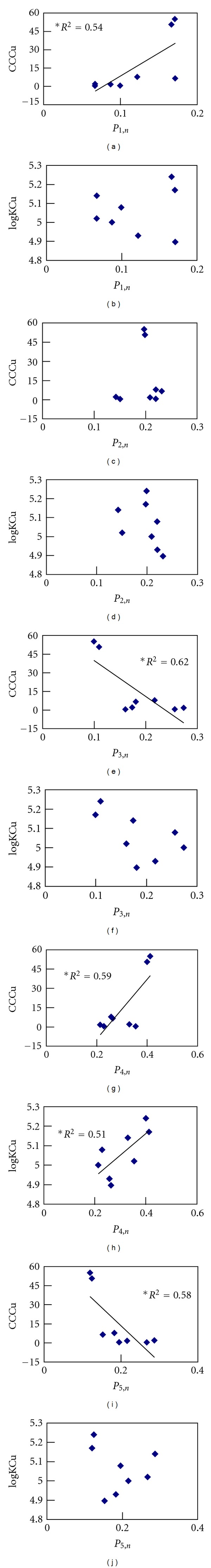
Relationships between percentages of fluorescence response of the five regions (*P*_*i*,*n*_) and complexing capacity (CC_Cu_) and the conditional stability constant (log*K*_Cu_) *Statistical significance value *P* < 0.05.

The high determinative coefficients (DCs) in Table 1 indicated that the experimental data fit well with the Ryan-Weber model [26]. The complexing capacity (CC_Cu_) is an indicator presenting the amount of active binding sites for complexation with metal ions in a unit mass of DOM. The CC_Cu_ of raw pig manure ranged from 50.6 to 55.0 mmol/g, which was far higher than that of raw cattle manure. After the composting process, the manure DOM featured much lower CC_Cu_ values in the range of 0.41 to 1.99 mmol/g. The increases in *P*_3,*n*_ and *P*_5,*n*_ confirmed that humification of manure DOM occurred during composting. Previous study has shown that carboxylic- and phenolic-type groups are two kinds of binding sites in humic acids and fulvic acids [30]. Plaza et al. [31] investigated that total acidic functional group contents including phenolic OH group and carboxyl group increased in cattle manure after composing. Caricasole et al. [16] also reported the increasing of phenolic, carboxylic, and carbonylic C occurred in DOM from domestic organic wastes after composting. However, there were negative linear relationships between CC_Cu_ and *P*_5,*n*_ (*R* ^2^ = 0.58, *P* < 0.01) or *P*_3,*n*_ (*R* ^2^ = 0.62, *P* < 0.01) shown in Figure 4. In contrast, significantly positive linear correlations were found between CC_Cu_ and *P*_4,*n*_ (*R* ^2^ = 0.59, *P* < 0.01) or *P*_1,*n*_ (*R* ^2^ = 0.54, *P* < 0.01). The results may suggest that raw manure DOM has higher CC_Cu_ than that of composted manure DOM that can be attributed to its more tyrosine-like organic compounds and soluble microbial byproduct-like materials. The remarkable diversity of Cu complex capacity may be attributed to the difference of protein-like materials in manure DOM. In surface water, fulvic acid and humic acid in DOM were accepted as main components which complexed with Cu [6]. However, Yamashita and Jaffé [7] reported that nonhumic substances such as amino acids are likely engaged in the Cu complexation in surface water. de Zarruk et al. [9] have also verified that the fraction with high proteinaceous fluorescence in vinasse formed the most DOM-Cu complexes. Thus, the binding of Cu to DOM was inhibited properly due to the degradation of protein-like materials during composting, particularly in DOM from composted pig manure.

The conditional stability constants (log⁡⁡*K*_Cu_) of Cu complexes with manure DOM were between 4.90 and 5.24 (Table 1), and the values are similar to those studies on surface water DOM [7]. The DOM of composted cattle manure with sawdust and corn stalk featured higher log⁡⁡*K*_Cu_ than that of raw cattle manure. On the contrary, pig manure DOM showed the decreasing trend after composting. The significantly positive linear correlation between log⁡⁡*K*_Cu_ and *P*_4,*n*_ can be observed in Figure 4 (*R* ^2^ = 0.51, *P* < 0.01). This may indicate, in part, that some functional groups in soluble microbial byproduct-like materials play an important role in determining the conditional stability constant log*K*_Cu_. Soluble microbial byproduct-like materials contain a large number of proteins and amino acids [20]. It has been reported that protein-like fraction of natural water DOM has the highest log⁡⁡*K*_Cu_ [23]. If it was also true for manure DOM, the decrease in the complex stability of pig manure DOM may be attributed to the degradation of large amounts of aromatic amino acid materials.

## 4. Conclusion

The composting process reduced the amounts of DOM in pig and cattle manure. A majority of the protein-like materials were decomposed, and new humic-like and fulvic-like components were repolymerized. Humic-like materials in composted DOM were mainly transformed from tryptophan-like organic compounds, whereas fulvic-like components were largely transformed from soluble microbial byproduct-like substances.

The complexing capacities of pig and cattle manure DOM decreased after composting, which can be attributed to the degradation of protein-like components. Furthermore, the degradation of protein-like components in pig manure reduced the stability constants of log⁡⁡*K*_Cu_. Our study suggests that the composting process might be a way to decrease the bioavailability, mobilization, and transport of manure DOM-Cu complexes, and lower the potential pollution risk to soil and ground water. 

## Figures and Tables

**Table 1:** Fitting parameters of Ryan-Weber model.

DOM samples^a^	Ex/Em (nm)^b^	*I*_Cu*L*_ (AU)^b^	CC_Cu_ (mmol/g)^b^	log⁡*K*_Cu_^b^	DC^b^
A1	276/306	260.98	50.60	5.24	0.992**
A71	315/412	79.04	1.99	5.14	0.998**
B1	276/306	296.51	55.00	5.17	0.987**
B71	315/412	91.00	0.41	5.02	0.996**

C1	305/412	39.90	6.52	4.90	0.997**
C46	310/408	46.48	0.53	5.08	0.998**
D1	310/414	81.69	7.78	4.93	0.996**
D46	310/420	53.71	1.62	5.00	0.998**

^a^DOM from treatment A (pig manure + sawdust) on days 1 and 71 (A1, A71), treatment B (pig manure + corn stalk) on days 1 and 71 (B1, B71), treatment C (cattle manure + sawdust) on days 1 and 46 (C1, C46), and treatment D (cattle manure + corn stalk) on days 1 and 46 (D1, D46).

^b^Ex/Em: selected wavelengths for the fluorescence titration; *I*_Cu*L*_: fluorescence intensity of Cu ion-saturated complex (arbitrary units: AU); CC_Cu_: complexing capacity (mmol/g); log *K*_Cu_: the conditional stability constant; DC: determinative coefficiency.

**Statistical significance value *P* < 0.001.
